# Changes in the Geographic Distribution of the Diana Fritillary (*Speyeria diana*: Nymphalidae) under Forecasted Predictions of Climate Change

**DOI:** 10.3390/insects9030094

**Published:** 2018-08-02

**Authors:** Carrie N. Wells, David Tonkyn

**Affiliations:** 1 Department of Biological Sciences, University of North Carolina at Charlotte, 9201 University City Blvd, Charlotte, NC 28223, USA; 2 Department of Biology, University of Arkansas at Little Rock, 2801 South University Ave., Little Rock, AR 72204, USA; dwtonkyn@ualr.edu

**Keywords:** *Speyeria diana*, butterfly, conservation, fragmentation, global warming, Maxent, WorldClim

## Abstract

Climate change is predicted to alter the geographic distribution of a wide variety of taxa, including butterfly species. Research has focused primarily on high latitude species in North America, with no known studies examining responses of taxa in the southeastern United States. The Diana fritillary (*Speyeria diana*) has experienced a recent range retraction in that region, disappearing from lowland sites and now persisting in two phylogenetically distinct high elevation populations. These findings are consistent with the predicted effects of a warming climate on numerous taxa, including other butterfly species in North America and Europe. We used ecological niche modeling to predict future changes to the distribution of *S. diana* under several climate models. To evaluate how climate change might influence the geographic distribution of this butterfly, we developed ecological niche models using Maxent. We used two global circulation models, the community climate system model (CCSM) and the model for interdisciplinary research on climate (MIROC), under low and high emissions scenarios to predict the future distribution of *S. diana*. Models were evaluated using the receiver operating characteristics area under curve (AUC) test and the true skill statistics (TSS) (mean AUC = 0.91 ± 0.0028 SE, TSS = 0.87 ± 0.0032 SE for representative concentration pathway (RCP) = 4.5; and mean AUC = 0.87 ± 0.0031 SE, TSS = 0.84 ± 0.0032 SE for RCP = 8.5), which both indicate that the models we produced were significantly better than random (0.5). The four modeled climate scenarios resulted in an average loss of 91% of suitable habitat for *S. diana* by 2050. Populations in the southern Appalachian Mountains were predicted to suffer the most severe fragmentation and reduction in suitable habitat, threatening an important source of genetic diversity for the species. The geographic and genetic isolation of populations in the west suggest that those populations are equally as vulnerable to decline in the future, warranting ongoing conservation of those populations as well. Our results suggest that the Diana fritillary is under threat of decline by 2050 across its entire distribution from climate change, and is likely to be negatively affected by other human-induced factors as well.

## 1. Introduction

Understanding how species distributions might shift with the changing climate is a critical component of managing and protecting future biodiversity. Hundreds of species in the United States and elsewhere have responded to the warming climate by shifting to higher latitudes or elevations [1,2,3,4]. Such range shifts have been documented in a number of taxa [5,6,7], including alpine plants [8], marine invertebrates [9], marine fish [10], mosquitoes [11], birds [12,13], and butterflies [1,14,15,16,17,18]. A number of species distribution models have been developed to predict the impacts of climate change on species distributions, including bioclimate envelope models, which are useful first estimates of the potential effects of climate change on altering species’ ranges [19]. Bioclimate envelope models work by identifying the climatic bounds within which a species currently occurs, and then delineating how those climatic bounds will shift under various future climate projections [20,21,22,23].

Most often, researchers are limited to presence-only occurrence data, requiring the use of indirect methods to infer a species’ climatic requirements [8,24,25]. One of the best performing models using presence-only data is maximum entropy modeling, or Maxent [26], which performs well even with low sample sizes typical of rare species [19,27,28]. Maxent works by comparing climate data from occurrence sites with those from a random sample of sites from the larger landscape to minimize the relative entropy of statistical models’ fit to each data set. Species distribution models such as Maxent have been criticized for being overly simplistic, because they do not incorporate external biotic factors such as species interactions [20,27,29]. However, such bioclimate envelope models have been used to project with reasonable accuracy whether species ranges will increase or decrease under a changing climate [19,30,31,32], which was the primary objective of this study.

*Speyeria diana* (Nymphalidae) (Cramer 1777) is a butterfly species endemic to the southeastern United States and is currently threatened across portions of its range. This species is of particular conservation interest because it has experienced a range collapse in recent decades resulting in an 800-km geographic and genetic disjunction between western populations in the Ouachita and Ozark Mountains and populations in the southern Appalachian Mountains, and has shifted to a higher elevation at an estimated rate of 18 m per decade [33]. This range contraction is consistent with the predicted effects of a warming climate, and might represent the first such documented case in the southeastern United States, though the region has experienced other environmental changes in recent decades as well [33]. Previous research using coalescent-based population divergence models dated the earliest splitting of the western population from the east at least 20,000 years ago, during the last glacial maximum [34]. In addition, recent geometric morphometric evidence from the wings of *S. diana* further support this long-term spatial and genetic isolation [35]. In light of these pieces of evidence, we used Maxent to model the future distribution of *S. diana* under several future climatic scenarios, in order to forecast how the range of the butterfly might shift under predicted conditions. Forecasts of large range reductions (over 50%), or small overlaps between current and future ranges (less than 50%), would suggest high vulnerability to climate change. Range reductions of any size in the western distribution would likely threaten those populations that are genetically isolated and adapted to relatively low dispersal, with the negative effects of genetic drift [34,35]. 

## 2. Methods

### 2.1. Study Species

The Diana fritillary, *Speyeria diana*, is a large and sexually dimorphic nymphalid butterfly, endemic to the southeastern United States. Adult males emerge in late May to early June, with females flying several weeks to a month later [36]. Once mated, each female can lay thousands of eggs singly on ground litter during the months of August and September in the vicinity of *Viola* spp., the larval host plant for all *Speyeria* [37]. After hatching, first instar larvae immediately burrow deep into the leaf litter layer of the forest floor, where they overwinter [38]. In spring, larvae feed on the foliage of freshly emerging violets. Adult Diana butterflies are often found along forest edges or dirt roads containing tall, conspicuous nectar sources such as milkweeds, butterfly bushes, or other large summer and fall composites [39,40,41,42]. While males begin to die off in late July, females may persist in large numbers, although somewhat cryptically, through October [42].

### 2.2. Distributional Dataset

We searched for all known records of *S. diana*, from publications, catalogued and uncatalogued specimens in public and private collections in the United States and Europe, online databases, contemporary field surveys by scientists and amateurs, and our own field surveys. We obtained distributional data from 1323 pinned *S. diana* specimens from 33 natural history museum collections in the United States and Europe (Table 1). Four hundred thirty-five additional records (1938–2012) were provided by the Butterfly and Moth Information Network and the participants who contribute to its BAMONA project. Our literature survey produced 153 records (1818–2011) across 54 U.S. counties (Table 2). We also collected 469 *S. diana* butterflies in our own field surveys (Table 3). Our dataset essentially represents a complete dataset of all publicly available records for the species, and is as comprehensive as for any taxon in the region [33]. For this reason, our dataset should be especially informative in creating an accurate bioclimate envelope for the species, as collection bias is a major consideration with ecological niche modeling [43,44].

### 2.3. Species Distributional Modeling

We developed species distribution models using the popular machine-learning algorithm for ecological modeling, Maxent [26]. Maxent estimates a species’ probability distribution that has maximum entropy (closest to uniform), subject to a set of constraints based on the sampling of presence-only data [45]. Because of the difficulty and impracticality of obtaining accurate absence data, presence-only data are most often used in species distribution modeling. In order to offset the lack of absence data, Maxent uses a background sample to compare the distribution of presence data along environmental gradients with the distribution of background points randomly drawn from the study area [46,47,48]. Locality data and the randomly sampled background points are combined with climatic data to predict the probability of the species’ occurrence within each raster grid cell. We used environmental climate data from WorldClim [49] at 30 arc-second resolution or approximately 1 km^2^ grid cells. Bioclimate variables and elevation layers were each clipped to the extent of North America using ESRI (Environmental Systems Research Institute) ArcMap 10.0, and data extracted to *S. diana* sample localities. Additionally, we collected the same types of locality data for three other species of North American butterflies (*Speyeria cybele*, *Speyeria idalia*, *Battus philenor*), which served as 5628 random background points for our models. We utilized these background data to minimize spatial bias in our modeling, as data represented by similar butterfly species can be used as pseudo-absence data with the same collection bias as our occurrence data, improving the accuracy of the model [50,51]. 

Climatic variables included 19 derived bioclimatic variables that describe annual and seasonal variation in temperature and precipitation, as well as elevation, averaged for 1950–2000 (Table 4). One concern when modeling species distributions is the strong correlation that occurs between multiple climate variables, which can significantly influence model predictions of species distributions [52]. To test for co-linearity, we performed spatial autocorrelation statistics between all pairs of the 19 bioclimate variables using ESRI ArcMap 10.0. We then selected the most biologically meaningful variable for each group of two or more variables with Pearson correlation coefficients higher than 0.7 (Table 4). This allowed us to reduce the number of bioclimate variables to the nine potentially most important ones, which were: Minimum Temperature of Coldest Month, Mean Temperature of Driest Quarter, Precipitation of Wettest Month, Precipitation of Driest Month, Precipitation of Driest Quarter, Isothermality, Mean Diurnal Range (Mean of monthly (maximum temperature—minimum temperature)), Temperature Annual Range, and Annual Precipitation, along with elevation (Table 4). These variables are typically considered to be important determinants of butterfly distributions, as they relate to life history traits. Butterflies are highly sensitive to weather and climate, particularly changes in temperature and rainfall [53]. For example, mean temperature of the coldest month is related to the overwintering survival of first instar larvae, growing degree days above 5 °C are regarded as a surrogate for the developmental threshold of the larvae, water balance corresponds to the moisture availability for the larval host and adult nectar plants, and the mean temperature of late summer ensures proper adult emergence and mating [54,55,56,57,58,59]. Temperature changes affect all aspects of butterfly life history, from their distribution and abundance [14,54], to their realized fecundity [60,61]. Changes in rainfall levels can influence butterfly larvae indirectly through changes in host plant quality, and generally rainfall is considered to be beneficial because it enhances host plant growth [62]. 

One concern when modeling species distributions is whether the occurrence records are spatially biased with respect to site accessibility (e.g., towns, roads, trails) [63]. To address this concern, we applied a spatial filter to remove all sampling points that were within 5 km of each other using ESRI ArcMap 10.0. The spatial filter resulted in 254 unique presence points for *S. diana* that were used in the final model. We first modeled the distribution of these 254 occurrences in present-day climate, and then projected the fitted species distribution under two future climate scenarios for the period 2040–2069 (hereafter referred to as 2050). Future climate scenarios were taken from two global circulation models (GCMs) obtained from www.worldclim.org; the community climate system model (CCSM) [64] and the model for interdisciplinary research on climate (MIROC) [65,66]. These GCMs differ in the reconstruction of several climatic variables and are well known to produce different outcomes for butterfly species [67,68]. For example, in hind-casting Mediterranean butterflies, the CCSM model projects narrower distributions at the last glacial maximum than does MIROC [65,66]. For each of these two GCMs, we considered two different representative concentration pathways (RCPs) [69,70,71,72,73], which are cumulative measures of human emissions of greenhouse gases from all sources expressed in Watts per square meter. These pathways were developed for the Fifth Assessment Report of the Intergovernmental Panel on Climate Change [67] and correspond to a total anthropogenic radiative forcing of RCP = 4.5 W/m^2^ (low) and RCP = 8.5 W/m^2^ (high) [72,73].

**Figure 1:**
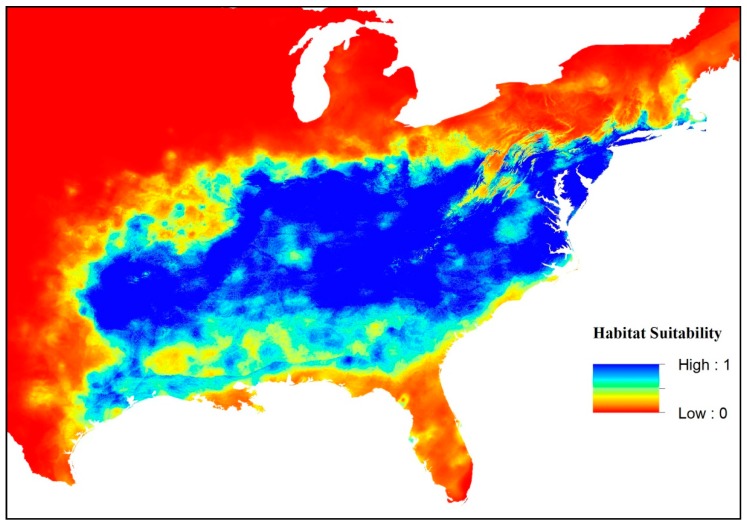
The present-day geographic distribution of *Speyeria diana*, with indices of habitat suitability as predicted by maximum entropy modelling (Maxent) under current climatic conditions (1950–2010).

We used Maxent’s default parameters [26,50] and a ten-fold cross-validation approach to further reduce bias with respect to locality data. This method divides presence data into ten equal partitions, with nine used to train the model, and the tenth used to test it. These partitions generate ten maps (one map per run), with each raster grid cell containing a value representing the probability of occurrence. These values were used to designate habitat suitability ranging from 0 (unsuitable habitat) to 1 (highly suitable habitat) (Figure 1). We averaged the resulting maps for the current climate, and for the two GCMs under RCP = 4.5 and RCP = 8.5. This method resulted in the production of a “low” and “high” average prediction for *S. diana* species distribution in 2050, represented with habitat suitability maps. We measured the goodness of fit for the models using the area under the curve (AUC) of a receiver-operating characteristic (ROC) plot [74]. We used criteria of Swets [75] and considered AUC values higher than 0.7 representative of model predictions significantly better than random values of 0.5 or less [26,27,74]. Because AUC has been recognized as a somewhat questionable measure of accuracy, especially when used with background data instead of true absences [74,76], we also calculated the TSS (true skill statistics), a threshold-dependent evaluation metric [76,77]. The relative importance of each variable’s contribution was assessed by sequential variable removal by Jackknife [26].

## 3. Results

Species distributional modeling resulted in “excellent” model fits for *Speyeria diana*, with a mean AUC = 0.91 ± 0.0028 SE, TSS = 0.87 ± 0.0032 SE for RCP = 4.5; and a mean AUC = 0.87 ± 0.0031 SE, TSS = 0.84 ± 0.0032 SE for RCP = 8.5 (Table 1). Annual precipitation explained the largest fraction of the distribution of *S. diana* under both RCPs (17.9%, RCP = 4.5; 19.4%, RCP = 8.5). Among the remaining bioclimatic variables, mean temperature of driest quarter had the next highest average percent contribution (10.3%, RCP = 4.5; 25.0%, RCP = 8.5), followed by minimum temperature of coldest month (20.1%, RCP = 4.5; 10.4%, RCP = 8.5), isothermality (7.3%, RCP = 4.5; 7.6%, RCP = 8.5), precipitation of wettest month (3.5%, RCP = 4.5; 3.9%, RCP = 8.5), precipitation of driest month (1.4%, RCP = 4.5; 5.4%, RCP = 8.5), precipitation of driest quarter (3.3%, RCP = 4.5; 2.4%, RCP = 8.5), Elev (1.5%, RCP = 4.5; 3.5%, RCP = 8.5), mean diurnal range (1.8%, RCP = 4.5; 2.8%, RCP = 8.5), and temperature annual range (1.6%, RCP = 4.5; 1.3%, RCP = 8.5) (Table 1).

**Figure 2:**
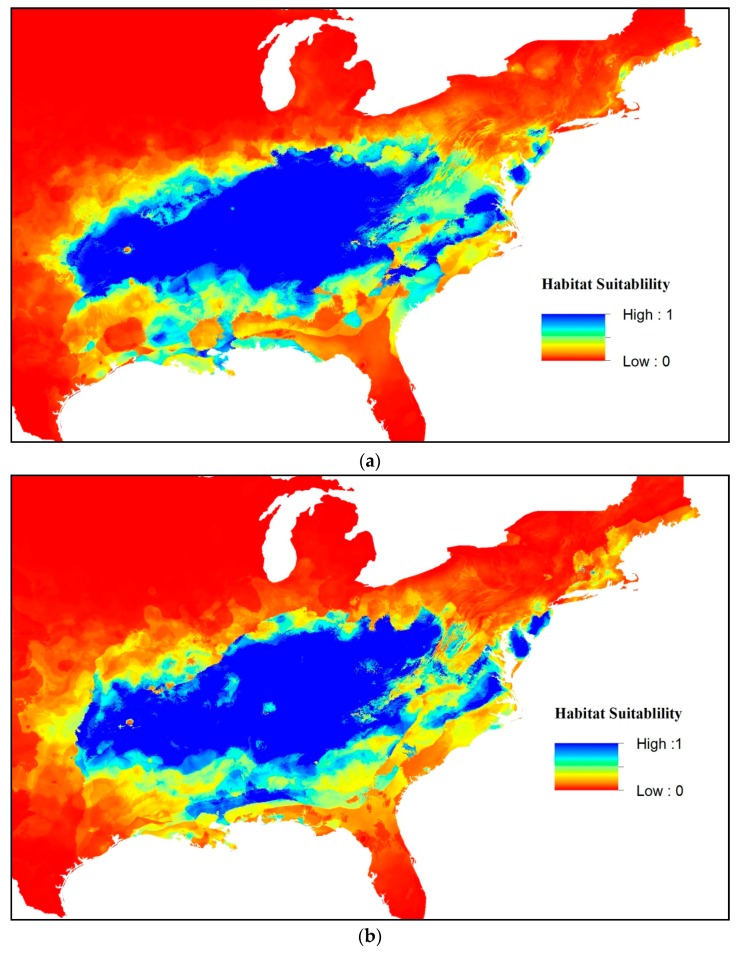
(**a**) Habitat suitability indices for the projected future distribution of *Speyeria diana* under the community climate system model (CCMA) and model for interdisciplinary research on climate (MIROC) representative concentration pathways (RCP) 4.5 climate change scenarios; (**b**) habitat suitability indices for the projected future distribution of *Speyeria diana* under the CCMA and MIROC RCP 8.5 climate change scenarios.

Modelling with Maxent under the selected climate-change scenarios predicted that habitat suitability would decrease for *S. diana* by 2050 (two-tailed paired *t*-tests comparing current Maxent values with those of 2050; all *p* < 0.01). The MIROC model resulted in more loss of suitable habitat than CCSM under both RCP scenarios (88.2% versus 92.4% of suitable habitat retained for RCP 4.5, and 90.2% versus 94.3% of suitable habitat retained for RCP 8.5 in CCSM and MIROC, respectively). Both climate models indicate that the loss of core distributional area is modest, with an average of 91.3% of present distributional areas retained. The most drastic reduction in habitat is apparent across the southern Appalachian Mountains (Figure 2).

## 4. Discussion

Our ecological niche models predicted that the amount of suitable habitat for *Speyeria diana* will decline substantially by the year 2050 across its entire distribution. Both CCSM and MIROC climate models predicted severe habitat loss and fragmentation in the southern Appalachian Mountains by 2050, with some range expansion predicted into higher latitudes in both eastern and western populations. High elevation habitat will be an important refuge for the species across the entire distribution, as the range of *S. diana* is already shifting to higher elevations at an estimated rate of 18 m per decade [33]. Recent evidence further suggests that some *S. diana* populations may already be adapting to high elevations, as *S. diana* female forewings from high elevation populations were found to be narrower than low elevation populations, indicating that these females may be more mobile than those from low elevations with wider forewings [35].

Unlike populations in the eastern distribution, the wing shape of western populations of *S. diana* appears to be better adapted for lower dispersal, which is in alignment with findings that western populations of *S. diana* are both spatially and genetically isolated [35]. Our models predicted that the southern edge of the highly suitable habitat in the west will recede by 2050; However, as was found in the southern Appalachian Mountains, the suitable habitat was predicted to expand in the higher elevations of the Ozark and Ouachita mountains of Arkansas. The genetic isolation of western populations may ultimately prevent them from adapting to higher elevations as successfully as populations in the eastern distribution of the species. If this is the case, lower elevation populations will be even more vulnerable to climate change than our models predict.

We would like to note that all ecological niche models should be used and interpreted with caution because of various sources of bias and error that result in inaccurate predictions [78]. Some have questioned the applicability of bioclimatic modeling at regional scales because of the somewhat coarse resolution [79]. However, we are confident that the size of our study area, and our uniquely extensive dataset, provide sufficient data to forecast climate-driven range shifts in *S. diana* with accuracy. Both global circulation models (CCCM and MIROC) were very closely aligned in their outcomes, indicating strong agreement between them. Climate is well understood to play a primary role in shaping the distributions of species [80], and we are confident in our overall findings that the suitable habitat for *S. diana* will decline and become increasingly fragmented by 2050.

## 5. Conclusions

These results highlight the importance of maintaining connectivity of the suitable habitat for *S. diana*, especially in the eastern populations that appear most vulnerable to increased fragmentation and loss of suitable habitat. These populations in the eastern distribution of *S. diana* harbor important genetic diversity that may become lost through genetic drift if these populations become small and isolated. The Ozark and Ouachita Mountains of Arkansas and Missouri appear to be least vulnerable to loss of suitable habitat from climate change, and therefore will be important for the future conservation of *S. diana* after 2050. As a result of the geographic and genetic isolation of the western populations, conservation of suitable habitat in the west is equally as important as in the east. Our climate models show that the 800-km disjunction across the center of the range of *S. diana* is not due to complete absence of suitable habitat, but more probably a result of the extensive habitat fragmentation regionally across the Ohio River Valley from agricultural land use change, and other human related factors that were not included in our models. We conclude that maintaining well-connected low and high elevation habitats across the entire distribution of *S. diana,* both now and into the future, will be necessary for this species, even under conservative forecasts of climate change.

## Figures and Tables

**Table 1:** Summary of *Speyeria diana* distributional data sources (adapted from Wells and Tonkyn 2014).

National Museums (N. American)	Location	No. of *S. diana*	Range of Specimen Dates	No. of Counties
Carnegie Museum of Natural History	Pittsburgh, Pennsylvania	142	1889–2000	26
National Museum of Natural History	Washington, DC	129	1907–2002	26
American Museum of Natural History	New York, NY	104	1921–1985	28
The Field Museum	Chicago, IL	98	1889–1995	23
California Academy of Sciences	San Francisco, CA	88	1886–2000	12
Georgia Museum of Natural History	Athens, GA	15	1935–1987	8
Cleveland Museum of Natural History	Cleveland, Ohio	6	1921–1965	6
Denver Museum of Nature and Science	Denver, Colorado	4	1939–1973	3
Mount Magazine State Park	Paris, Arkansas	4	1997	1
**National History Museums (European)**				
British Natural History Museum	London, UK	31	1777–1989	17
Paris Muséum national d’Histoire naturelle	Paris, France	8	1890	1
Oxford Museum of Natural History	Oxford, UK	4	1937–1971	4
Zoölogisch Museum Amsterdam	Amsterdam, The Netherlands	4	1884–1921	3
Naturalis Biodiversity Center	Leiden, Netherlands	4		
Royal Ontario Museum	Ontario, Canada	3	1933–1968	3
**University Collections**				
University of Florida	Gainesville, Florida	409	1900–2007	43
University of Michigan	East Lansing, Michigan	66	1909–1985	13
Clemson University	Clemson, South Carolina	43	1926–1978	5
Peabody, Yale University	New Haven, Connecticut	29	1904–1961	8
University of Missouri	Columbia, Missouri	29	1886–1980	8
University of Wyoming	Laramie, Wyoming	13	1955–1979	4
University of Arkansas, Little Rock	Little Rock, Arkansas	12	2005–2007	5
University of California, Berkley	Berkley, California	12	1926–1981	6
University of Nebraska	Lincoln, Nebraska	14	1954–2003	7
North Carolina State University	Raleigh, North Carolina	10	1904–1964	9
University of Arkansas, Fayetteville	Fayetteville, Arkansas	10	1977–1994	5
Virginia Polytechnic Inst	Blacksburg, Virginia	8	1911–1977	1
Louisiana State University	Baton Rouge, Louisiana	7	1984–1988	1
University of Wisconsin	Madison, WI	5	1926–1951	2
College of Charleston	Charleston, South Carolina	4	2008	2
West Virginia University	Morgontown, West Virginia	3	1977–1995	2
Furman University	Greenville, South Carolina	3	1929–1990	3
Dalton State College	Dalton, Georgia	2	2001	1
**State Agencies, online databases, listserves, individuals, and organizations**				
Field Surveys		469	1995–2012	46
Butterflies and Moths of America (BAMONA)		435	1938–2012	39
North Carolina 19th Approximation (http://149.168.1.196/nbnc/)		276	1938–2011	31
West Virginia Divisions of Natural Resources (wvdnr.gov)		204	1978–1999	11
Literature survey		153	1818–2011	54
Kentucky Dept. of Fish and Wildlife Resources (fw.ky.gov)		146	1936–2006	21
NABA annual count data (naba.org)		103	1999–2010	27
Georgia Dept. of Natural Resources (gadnr.org)		77	1994–2001	15
Global Biodiversity Information Facility (GBIF)		75	1974–2004	49
North Carolina Natural Heritage Program (nchp.org)		69	1989–2003	21
The Lepidopterists’ Society (lepsoc.org)		50	1973–2008	25
All Taxa Biodiversity Inventory (ATBI) (dlia.org/atbi)		46	1936–2007	4
Carolina Butterfly Society (CBS)		44	2001–2009	5
Carolinaleps		41	2007–2009	9
Washington Area Butterfly Club		29	2007	1
Oklahoma Leps		21	2005–2009	5
Insect.net		21	2007–2009	9

**Table 2:** Summary of literature referencing the distribution of *Speyeria diana* (adapted from Wells and Tonkyn 2014).

Reference	Location	Date of Record(s)	Description
Cramer & Stoll 1775	Jamestown, Virginia	1775	holotype; male described by Pieter Cramer
Blatchley 1859	Vanderburgh County, Indiana	1850s	first record from Indiana, most northern record
Edwards 1864	Kanawha, West Virginia	20–31 August 1864	first description of female, took over 30 specimens
Edwards 1874	Coalburgh, West Virginia	August, September 1873	description of rearing *Argynnis* larvae
Aaron 1877	Tennessee/North Carolina	1877	populations are ample along Blue Ridge
	Kentucky	1877	locally abundant populations
Strecker 1878		1878	West Virginia, Georgia, Kentucky, Tennessee, Arkansas
Thomas 1878	Kentucky, Arkansas, southern Illinois	1878	common in Kentucky & Arkansas
Fisher 1881	Illinois	1880	present in southern Illinois
Holland 1883	Salem, North Carolina	1858–1861	described as “first pinned female specimen”
Edwards 1884	southern Ohio	1880s	first description in Ohio
Hulst 1885	Waynesville, North Carolina	1882	locally abundant populations
	Warren Springs, North Carolina	1882	very common along the French Broad River
Blatchley 1886	Evansville, Indiana	early 1900s	locally abundant populations
French 1886	eastern United States	1886	W. Virginia to Georgia, Southern Ohio to Illinois, Kentucky, Tennessee, Arkansas
Hine 1887a, b	Medina County, Ohio	9 August 1887	single worn male, northernmost record in OH
Kingsley 1888	Virginia	1887	*Argynnis diana* is described as the handsomest insect found in the United States
Scudder 1889	southeast United States	1880s	*Semnopsyche diana;* an inhabitant of hilly country of the south, 38th parallel of latitude, taken as far west as Missouri and “Arkansaw”
Skinner & Aaron 1889	Pennsylvania	1880s	stray individual found in Pennsylvania
Dixey 1890	eastern United States	1889	description of *Argynnis diana* wing spot pattern
Blatchley 1891	Illinois	1890s	female specimen from northern Danville, IL
Skinner 1896	southern Illinois	1890s	Diana specimens from southern Illinois are larger than those further east
Holland 1898	southern United States	1890s	in two Virginias and Carolinas, northern Georgia, Tennessee, Kentucky, occasionally in southern Ohio and Indiana, and in Missouri and Arkansas; the most magnificent and splendid species of the genus
Snyder 1900	Clay County, Illinois	1900	northern limit of *S. diana* in Illinois
Strecker 1900	Missouri	1853	pair captured in copula, very early female
Maynard 1901			habitat is West Virginia to Georgia, southern Ohio to Illinois, Tennessee, and Arkansas
Sell 1916	Greene County, Missouri	22 August 1900	southeast of Springfield
Smyth 1916	southeast United States	1880–1916	Asheville, Brevard, North Carolina, Caesar’s Head, South Carolina, Montgomery, Washington and Giles Counties, Virginia
Wood 1916	Camp Craig, Virginia	August 1914	describes female color variation
Murrill 1919	Virginia	1919	Poverty Valley
Holland 1931		1930s	The Virginias and Carolinas, northern GA Tennessee, Kentucky, occasionally in southern OH, Indiana, and in Missouri and Arkansas
Knobel 1931	Hope, Arkansas	1930	from Mrs. Louise Knobel
Kite 1934	Taney County, Missouri	31 July 1925	male and female reported
Clark 1937	Virginia	1930s	ranges from Bath County, Virginia to FL east almost to tidewater, and west to Illinois and Arkansas
Clark & Williams 1937	Virginia	late 1800s–1935	Bath, Alleghany, Giles, Bland, Dickenson, Smyth, Patrick, Montgomery & Washington Counties
Allen 1941	West Virginia	1940	Pocahontas County, west to Kanawha and Lincoln Counties; abundant in Jefferson NF (Monroe County), Babcock State Park (Fayette County), and Fork Creek Wildlife Management Area (Boone County)
Chermock 1942	Conestee Falls, North Carolina	summer 1941	southern. Ohio and West Virginia, through the Appalachian mountains into Georgia and South Carolina, most abundant in mountains south of Great Smoky Mountains National Park
Bock 1949	Cincinnati, Ohio	1947	author collects hundreds of specimens from North Carolina mountains; gone from Indiana and Ohio
Clark & Clark 1951	Southern Illinois	early 1900s	
	Chesterfield County, Virginia	1930	last known county record
	Northampton County, Virginia	1930	last known county record
Klots 1951	Brevard, North Carolina	1950	in large numbers along roadsides; Chiefly in mountains and piedmont, W. Virginia s. to Georgia, w. to southern Ohio, Indiana, Missouri, and Arkansas
Mather & Mather 1958	Madison Parish, Louisiana	1958	record is a stray individual
Evans 1959	Smoky Mountains of Tennessee	September 1957	identification of an unknown *S. diana* larva
Curtis & Boscoe 1962	Buncombe County, North Carolina	27 June 1962	collecting record near Asheville
Hovanitz 1963	Salem, Roanoke County, Virginia	13 June 1937	comprehensive distribution data
Ross & Lambremont 1963	Louisiana	1950s	stray record from Mather & Mather 1958
Masters 1968	Newton County, Missouri	1960s	locally very common
Masters & Masters 1969	Perry County, Indiana	15 July 1962	last record known from Indiana
Shull & Badger 1971	Indiana	1971	no longer resident in Indiana
Harris 1972	Georgia	1972	summarizes historic reports from White, Union, Fannin, Habersham, Rabun Counties
Irwin & Downey 1973	Vermilion County, Illinois	20 August 1960	female, last known Illinois record
	Southern Illinois	1880	Illinois natural history survey
Howe 1975		1950s	extirpated from type locality, Jamestown
	Kentucky, West Virginia	1970s	species is scarce in Kentucky and West
	Virginia		
	Georgia	1970s	not uncommon in northern Georgia
	Ceasar’s Head, South Carolina	1970s	stable populations, not uncommon
Nelson 1979	Ozark plateau of Oklahoma	1969	only found in eastern counties
Schowalter & Drees 1980	Poverty Hollow, Virginia	1973, 1978	field-captured and lab-reared *S. diana* gynandromorphs described in detail
Pyle 1981	eastern United States	1980s	has decreased its range because of forest loss, common in the Great Smoky Mountains
Hammond & McCorkle 1983	Virginia & Tennessee	1975–1978	Appalachian populations are expanding
Opler 1983	eastern United States	1980s	some populations under decline
Opler & Krizek 1984		1950s	extirpated from Virginia Piedmont and coast
		1800s	extirpated from Ohio River valley
Shuey et al. 1987	Cincinnati, Ohio	1900s–1930	eliminated by deforestation by early 1900s
Shull 1987	Indiana	late 1800s	occurs in mountains and piedmont of West Virginia south to Georgia, west to southern Ohio, Indiana, Missouri, and Arkansas
Watson & Hyatt 1988	Tennessee	1980s	resident species of northeastern Tennessee
Kohen 1989	Cumberland, Kentucky	July 1984	aberrant male on milkweed
Cohen & Cohen 1991	Bath County, Virginia	1990	George Washington National Forest
	Montgomery County, Virginia	1990	photograph of pair in copula
Krizek 1991	western Virginia	11 July 1991	males preferred nectar over horse manure
Adams 1992	Fannin County, Georgia	28 August 1992	female netted by Irving Finkelstein
Opler & Malikul 1992	eastern United States	1992	central Appalachians west to Ozarks, formerly Atlantic coastal plain of Va., NC, and Ohio River Valley, rich forested valleys
Skillman & Heppner 1992	Coopers Creek WMA Georgia	10 June 1988	Gynandromorph specimen found in n. GA
Carlton & Nobles 1996	Arkansas, Missouri, Oklahoma	1819–1995	survey of Interior Highlands
Allen 1997	West Virginia	1997	ranges from Virginia and W. Virginia south to northern Georgia and Alabama. A small population persists in Ozark Mountains of Arkansas and Missouri
Ross 1997	Coweeta Forest, North Carolina	1990, 1996	classified as uncommon, 2–5 individuals sighted
Ross 1998	Mount Magazine, Arkansas	30 June 1993	photograph of male, locally abundant
	Mount Magazine, Arkansas	20 August 1992	photograph of female, locally abundant
Glassberg 1999	eastern United States	1999	formerly throughout Ohio River Valley and southeastern Virginia and northwest N.C
Moran & Baldridge 2002	Arkansas, Missouri, Oklahoma	1997–1999	22 counties inhabited, Arkansas expanding
Scholtens 2004	Oconee County, South Carolina	2002	present in Sumter National Forest
Cech & Tudor 2005		2000s	locally common in mountain colonies, s. W. Virginia to n. GA; also e. AL/KY, Ozarks
Vaughan & Shepherd 2005	Red List species profile	2005	core of species distribution is in the southern Appalachians from central Virgina and W. VA through the mountains to northern Georgia and Alabama. Also in Ozarks of Missouri, Arkansas, and eastern Oklahoma
Adams & Finkelstein 2006	Fannin County, Georgia	12 October 2006	lots of aggregating females flying late
Rudolph et al., 2006	Ouachita Mountains, Arkansas	1999–2005	feeding records by month sites
Spencer 2006	Arkansas	2006	uncommon to locally common in colonies Scattered throughout the Interior Highlands Coastal Plain
Campbell et al., 2007	North Carolina	17 June 2004	at least four males visiting flowering sourwood
Ross 2008	Mount Magazine, Arkansas	2008	description of Mount Magazine State Park
Wells et al., 2010	Mount Magazine, Arkansas	2009	copulating pair photographed
Wells et al., 2011	Georgia, North Carolina, Tennessee	2009	females collected for rearing trial

**Table 3:** Field-sampled *Speyeria diana* (2006–2009). Records are provided to the level of county. All voucher specimens are held at the Clemson University Arthropod Collection (adapted from Wells and Tonkyn 2014).

State	County	Ecoregion	# *S. diana* (m/f)	Survey Dates
Arkansas	Benton	Ozark Plateau	7 (7/1)	12–14 June 2007, 22–23 June 2009
	Carroll	Ozark Plateau	9 (7/2)	15–16 June 2007, 23–24 June 2009
	Boone	Ozark Plateau	2 (2/0)	16 June 2007
	Faulkner	Arkansas River Valley	5 (5/0)	18–20 June 2006, 20 June 2007, 16 June 2008, 3–6 August 2009
	Conway	Arkansas River Valley	15 (11/4)	22 June 2007, 26 June 2008, 5 August 2009
	Pulaski	Arkansas River Valley	4 (2/2)	28 August 2009
	Logan	Arkansas River Valley	37 (29/8)	20–24 June 2006, 21–24 June 2007, 1–3 August 2009
	Montgomery	Ouachita Mountains	12 (7/5)	31 July 2008, 1–3 September 2009
	Polk	Ouachita Mountains	5 (1/4)	1–3 September 2009
	Saline	Ouachita Mountains	8 (7/1)	14 June 2008, 18 June 2009
Oklahoma	Leflore	Ouachita Mountains	3 (0/3)	30 August 2009
Georgia	Fannin	Blue Ridge Mountains	26 (17/9)	12–13 July & 1 August 2006, 12 July 2007, 22 June & 20 July 2008
	Rabun	Blue Ridge Mountains	8 (2/6)	7 September 2008, 29 August 2009
	Union	Blue Ridge Mountains	14 (6/8)	29 July 2007, 15 June & 5–7 August 2008,
North Carolina	Ashe	Blue Ridge Mountains	4 (4/0)	22–23 June 2007
	Buncombe	Blue Ridge Mountains	13 (8/5)	27 July 2006, 30 July 2007, 9 August 2008
	McDowell	Blue Ridge Mountains	15 (10/5)	9 September 2007, 24 June 2008, 30 June, 11 September 2009
	Transylvania	Blue Ridge Mountains	24 (19/5)	5 June 2006, 16 July & 5 September 2007, 14 June 2008, 26 June 2009
	Watauga	Blue Ridge Mountains	7 (5/2)	30 May & 9 June 2006, 25 July 2008, 19 September 2009
South Carolina	Greenville	Blue Ridge Escarpment	12 (7/5)	31 June 2006, 27–29 July 2007, 1 September 2008, 8–13 September 2009
Tennessee	Blount	Great Smoky Mountains	42 (33/9)	1–26 June 2007, 1–28 June & 20–29 August 2008, 1–15 September 2009
	Sevier	Great Smoky Mountains	33 (25/8)	1–26 June 2007, 26–29 June 2008, 5 June-26 September 2009
	Carter	Appalachian Mountains	57 (35/22)	5–9 June & 5–11 July 2006, 30–31 May 2007, 29–30 August 2008
	Sullivan	Appalachian Mountains	36 (25/11)	13–16 July 2006, 20–22 July 2007, 5 August, 18–20 September 2009
Virginia	Montgomery	Appalachian Mountains	21 (14/7)	3–7 July 2007, 2–4 July 2008

**Table 4:** Elevation plus the 19 bioclimate variables from the WorldClim dataset (Hijmans et al., 2005) collapsed into groups of highly correlated variables (Pearson’s correlation coefficient, *r* ≥ ±0.70), and their corresponding contribution to the Maxent model. The ten variables kept in the final model are bold and highlighted in grey. The community climate system model (CCCM) and model for interdisciplinary research on climate (MIROC) global circulation models are shown under representative concentration pathways (RCPs) 4.5 (low) and 8.5 (high), as predicted by the Intergovernmetnal Panel on Climate Change (IPCC) 5th report on climate. AVG—average; AUC—area under curve.

Bioclimate Variables	Abbreviation	% Contribution					
		CCCM-45	MIROC-45	AVG	CCCM-85	MIROC-85	AVG
Annual Mean Temperature	Bio 1	4.4	0.7	2.5	0.5	1.4	0.96
Max Temperature of Warmest Month	Bio 5	0.6	1.7	1.2	1.4	0.8	1.1
Min Temperature of Coldest Month	**Bio 6**	3.9	36.3	20.1	2.6	3.3	10.4
Mean Temperature of Wettest Quarter	Bio 8	14.1	10.2	12.2	4.0	16.8	2.6
Mean Temperature of Driest Quarter	**Bio 9**	15.5	5.1	10.3	30.2	19.8	25.0
Mean Temperature of Warmest Quarter	Bio 10	0.5	0.8	0.7	0.1	0.3	0.2
Mean Temperature of Coldest Quarter	Bio 11	0.8	12.5	11.9	3.3	1.5	2.4
Precipitation of Wettest Month	**Bio 13**	3.7	0.2	3.5	2.0	5.8	3.9
Precipitation Seasonality	Bio 15	6.0	3.7	4.9	8.7	2.7	5.6
Precipitation of Wettest Quarter	Bio 16	0.8	0.6	0.7	0.2	0.9	0.6
Precipitation of Warmest Quarter	Bio 18	1.1	0.3	1.0	1.9	1.0	1.5
Precipitation of Driest Month	**Bio 14**	0.9	1.6	1.4	2.7	8.0	5.4
Precipitation of Driest Quarter	**Bio 17**	4.2	2.3	3.3	2.2	2.6	2.4
Precipitation of Coldest Quarter	Bio 19	0.1	0.2	0.2	0.2	1.7	0.9
Elevation	**Elev**	2.0	1.0	1.5	4.9	2.0	3.5
Isothermality (BIO 2/BIO 7) (*100)	**Bio 3**	11.0	3.5	7.3	8.5	6.6	7.6
Temperature Seasonality (standard deviation *100)	Bio 4	6.4	1.0	3.7	0.0	4.2	2.1
Mean Diurnal Range (Mean of monthly (max temp—min temp))	**Bio 2**	0.6	3.0	1.8	2.0	3.6	2.8
Temperature Annual Range (BIO 5–BIO 6)	**Bio 7**	1.2	1.9	1.6	1.5	1.0	1.3
Annual Precipitation	**Bio 12**	22.3	13.4	17.9	22.9	15.9	19.4
AUC		0.86	0.96	0.91	0.87	0.86	0.87
